# Head-mounted display versus computer monitor for visual attention screening: A comparative study

**DOI:** 10.1016/j.heliyon.2023.e16610

**Published:** 2023-06-01

**Authors:** Tanja Baertsch, Ying-Yin Huang, Marino Menozzi

**Affiliations:** a Department of Health Sciences and Technology, ETH Zürich, Zurich, 8092, Switzerland; b Department of Industrial Engineering and Management, National Taipei University of Technology (Taipei Tech), Taipei, Taiwan

**Keywords:** Cognitive workload, Detectability, Head-mounted display, Occupational health practice, Visual attention

## Abstract

Visual attention is crucial to many tasks during working. When it is impaired, the risk of occupational accidents is increased. A potential accident prevention would be the tracking of employees' attentional states to construct break regimes. There is a promising visual attention test administered on a computer monitor (CM) that has several advantages over widely used continuous performance tests in detecting inattentiveness in occupational environments. However, as the setup with a CM is impractical for the use in particular working environments (e.g., lack of space or disturbing exposure to light), the test was implemented into a head-mounted display (HMD). This study aimed to investigate whether the HMD version of the test is a suitable alternative to the CM version. For this purpose, participants (N = 30; 20–29 y) performed both tests. The performance on the HMD was significantly lower than on the CM. Moreover, the performances were compared with normative data recorded with a CM in a previous study. These data significantly differ from the data recorded with the CM in the present study. This emphasizes the importance of a standardized test environment, which could be provided by an HMD. Conclusively, this study revealed that the new VR tool, based on a previous test designed to assess visual skills in a complex visual environment, exhibited good psychometric property regarding the reliability. In additional, no problems were revealed regarding the functionality and usability of the HMD.

## 1 Introduction

Typical visual scenes in daily life are complex and inundated with a large amount of perceptual information. Attention is one of the most fundamental cognitive functions used to cope with these complex scenes. Attention mechanisms allow concurrent selection and inhibition of information, thereby preventing information overload and reducing complexity [1]. According to Posner and Peterson [2], three networks control this mechanism: alerting, executive control, and orienting. The alerting network is a state of enhanced vigilance and readiness to respond to incoming stimuli. The executive network is comprised of top-down processes involved in inhibitory function for resolving conflict among possible responses. The attentional network of orienting is characterized as selecting information from sensory inputs by shifting processing resources to a particular location [2,3]. Where the function of these networks are not intact, workers generally have difficulty obtaining and processing visual information from the environment, thereby increasing the likelihood that errors in performing a task will occur or the risk of occupational accidents will increase because potential hazards are overlooked [4,5]. One way to ensure that people only work when there is a sufficiently high level of attention to perform the work can be achieved by regularly tracking their attention state and deriving a break regime from it. For the monitoring of the attention level, an objective assessment tool is required to measure attention capacity. This measurement could prevent working at low attention levels, and thus reduce the risk of accidents.

A widely used type of test to assess attention is continuous performance tests. These are objective tools for measuring attention, distractibility, inhibition capacity, and response speed [1]. Over time, continuous performance test presentations have evolved, changing stimulus presentation formats (audio/visual), stimulus types (geometrical figures, letters, numbers, spoken words, etc.) frequencies, and presentation durations. There are various tests based on computerized continuous performance test paradigms, varying in their specificity and sensitivity to diminished attention [6]. Common characteristics of those are the involving of sequential presentation of stimuli, e.g., letters or numbers, over a prolonged period of time, whereby the task is to attend to and respond to particular target stimuli presented alongside distracting stimuli. A widely used continuous performance test is, for example, the d2 test. It is a simple search task for targets embedded among distractors [7]. Another popular attention test is the psychomotor vigilance task, in that a button must be pressed as soon as a light randomly flashes on a uniform background [8]. An advance in capturing the complexity of visual tasks in real-world environments was made by the useful field of view (UFOV) test [9]. The UFOV test assesses various aspects of attention on a monochrome background on a screen. The test measures the abilities to perform a central visual identification task, to divide attention between central and peripheral stimuli, and to select peripheral stimuli among distractions [10]. There exists a variety of other attention tests [6]. However, when considering the use of a visual attention test to assess whether the attention capacity is sufficient for working safely in an occupational environment, it is crucial to use a test with ecological validity. This means that the design of a test should match the user’s real work context. The importance of the ecological validity was emphasized by Brennan et al. [11]. Results from search tasks conducted in real-world environment differed from results obtained from analogue experiments performed in a laboratory environment. The work environment is usually highly complex, and attention must usually be allocated not to a specific point in the visual field, but to the entire visual field. In addition, in many situations during working, not only simple responses to an event are required, but rather choice responses. Conclusively, a test assessing workers' attention level should optimally be a choice-reaction task that requires a response to a target stimulus appearing across the entire visual field alongside distracting stimuli on a complex background. As Menozzi et al. [12] could not find any appropriate test that met the requirements for use in occupational health practice in order to assess visual skills in a complex visual environment, they developed a new computerized test. The test assesses visual attentional performance at a moderate level of difficulty in the context of a perceptual process which requires constant attention over several minutes. The performance of recognizing and processing visual information is evaluated using a visual detection task against a complex background. Target detection includes recognizing the digit ‘3’ in a 6-digit number that briefly flashes either in the left, central, or right visual field. The test is originally developed to differentiate the low performers from the rest of the population by setting the presentation time of the stimuli at 300 ms, resulting in high levels of attentional skills that are not accurately captured. However, if attention should be effectively screened in occupations that require evaluated attentional skills, the stimuli presentation time can be reduced to 200 ms as Huang et al. [13] showed in their study. Thus, depending on attentional demands of a work, the difficulty level of the test can be adjusted. The test is performed on a computer monitor (CM) and a computer mouse is used to respond to a stimulus. In Menozzi et al.’s study [12], the setup of the attention test is explained in detail. In addition, normative data for the test were computed in the study using 150 data sets of participants with an age ranging between 15 years and 67 years who reported not taking drugs.

Although the visual attention test of Menozzi et al. [12] has good prerequisites for an ecological valid measurement of workers' attention level, its application is not optimal for all occupational environments. In some workplaces, the setup can be impractical, as the computer and the display cannot be installed due to lack of space or energy or difficult environmental conditions, such as disturbing exposure to light or extensive visual distractions.

To enable a more practical application, the visual attention test was programmed for use in a head-mounted display (HMD) in the scope of this work. To our best knowledge, there are currently no similar tools for assessing attentional capacity in workers. Therefore, it is important to analyze the effectiveness of this Menozzi et al.’s test [12] in the HMD for measuring attention performance in occupational environments. Virtual environments have already found use in several areas, such as engineering, education, and medicine [14]. The use of the test in the HMD offers several advantages but also some disadvantages over the CM. The next subchapter discusses the advantages and disadvantages in more detail.

### 1.1 Advantages and disadvantages of using head-mounted displays compared to using computer monitors to measure visual attention performance

The environment can be fully controlled during the testing by separating the visual stimuli from the external environment, ensuring a higher level of internal validity as the environmental bias is better controlled. This allows a standardization of visual presentation across varying environmental conditions by standardizing the entire visual field (e.g., color, luminance, and viewing distance) and by suppressing environmental distractions, which increases user engagement [15]. Distractions in the environment (whether auditory, visual, or physical) have been shown to cause short interruptions in attention [16]. Therefore, it can be expected that users wearing an HMD are less prone to distraction as they are isolated from external visual stimuli compared to sitting in front of a CM [17]. However, the present study was conducted in a low-distractive laboratory, so we assume that visual attention performance would not differ significantly between the two test environments. Another factor that can lead to distraction and affect the internal validity is the presence of other people, which likely to be the case in occupational environments. The presence of other people might affect test performance, as shown by a well-studied theory in social psychology called social facilitation and inhibition. Social facilitation refers to the tendency of people to accomplish simple tasks faster when they are accompanied by others, whereas social inhibition refers to the tendency to perform complex tasks poorly when others are present. An explanation for this is that the presence of others increases arousal level, resulting in enhanced or impaired performance depending on task complexity. Another explanation might be the self-presentation theory which posits that people’s presence motivates one to perform well, leading to better performance regardless of task difficulty. It is when the performer is embarrassed or distracted by their poor performance on complex tasks that the distinction between easy and complex tasks is made [18]. In working environments where many people are present, an effect of social inhibition on attention performance is expected. However, in the present study, no influence of social inhibition was expected because only the investigator—who did not sit directly beside the participant—and the participant were present in the experimental room. Therefore, it was likely that participants did not feel the presence of other people during either test.

Despite numerous important advantages of using HMDs for measuring attention purposes, there are also some limitations. Several studies showed that HMDs can induce motion sickness, of which simulator sickness and cybersickness are subtypes. Cybersickness is also known as virtual reality (VR) sickness [[19], [20], [21], [22], [23]]. Negative symptoms associated with HMDs are, for example, headache, nausea, blurred vision, difficulty focusing, eye fatigue, and eye strain [20,24,25]. Lee et al. [21] demonstrated in an eye-tracking experiment that the center-bias effect—when gaze fixations are biased towards the center of the scene—became stronger as the degree of VR sickness increased. However, Bahit et al. [26] came to a contradictory conclusion. They examined the effect of cybersickness on visual attention during day-night driving condition. Results indicated that severe cybersickness symptoms reduced visual attention as evidenced by lost focus at the center part of the computer scene, albeit only after sleep deprivation. Whereas, Wibirama et al. [27], who investigated the effect of sleep deprivation and day-night difference on visual attention, did not find any correlation between motion sickness and visual attention. Conclusively, the effect of sickness induced by VR on visual attention is not consistent [28]. However, since no pronounced sleepiness is expected in participants of the present study, we hypothesize that attentional performance is better with the CM than with the HMD due to expected higher sickness in the HMD environment. Whether or not motion sickness affects visual attention, the test must be designed and performed so that the likelihood of sickness occurring is minimized. Possible side effects can be alleviated by following some guidelines, such as reducing high acceleration movements in VR and reducing display field of view (FOV) [29]. Moreover, vergence-accommodation conflict is one of the main causes of eye strain and eye fatigue in HMDs, caused by a mismatch between perceived and virtual depth. Eyes converge and accommodate depending on the distance to a viewed object in order to obtain a clear vision. To obtain and maintain the viewed object in the fovea, eyes converge to look at the object and accommodation changes the eye’s lens. The accommodation is fixed in the HMD, which causes eye fatigue as accommodation and vergence are linked [20]. Symptoms of acute visual impairment may increase the likelihood of incorrect stimuli detection [30,31]. Studies demonstrated, for example, that reduced acuity significantly influences performance on neuropsychological tests with detailed and low-contrast items [30,31]. Kempen et al. [30] showed that blurred vision decreased performance on the Visual Form Discrimination test, which measures the ability to discriminate between complex visual configurations. Bertone et al. [31] investigated the effect of different levels of acuity loss on neuropsychological test performance by simulating blurred vision. Blurred vision affected the performance that leads to the assumption that the precision of the cognitive assessment (i.e., attention) are significantly biased when visuo-sensory abilities are not optimal. We, therefore, hypothesize that the attentional performance is better in the CM environment than in the HMD environment, where visuo-sensory abilities are expected to be more impaired. An overview of advantages and disadvantages of HMDs compared to CMs are presented in Table 1.

**Table 1:** Advantages and disadvantages of using HMDs compared to using CMs.

Advantages	Disadvantages
• Standardizing entire visual field o Isolation from external visual stimuli o Suppression of environmental distractions o Always the same distance from eyes to display • Reduced risk of social inhibition on the task performance • Practical application in occupational environment (does not require much preparation time, space, or controlling of environmental influences)	• Risk of VR sickness

Although HMD VR technology has some disadvantages, it has already been used as an alternative tool in scientific research on the human information processing system. In the following, related work on the use of VR in neuropsychological assessments are discussed.

### 1.2 Related work on neuropsychological assessments in virtual reality

There is evidence in favor of the use of VR measures in neuropsychological assessments. Studies have demonstrated sufficient test-retest reliability of HMD, which is a fundamental requirement for reliably assessing attentional performance [1,20,32]. Foerster et al. [20,32] conducted two studies to investigate whether commercially available HMDs are capable of reliable neuropsychological assessment. The test-retest reliabilities of several components of visual selective attention and processing capacity were assessed with the HTC Vive [20] and the Oculus Rift [32]. Reliabilities resulting from administering the test using an HMD and on a cathode ray tube display were compared. The results revealed that visual selective attention can also be recorded with a commercially available HMD (HTC Vive, Oculus Rift) as reliably as with a standard cathode ray tube display. Climent et al. [1] also showed that VR tools designed to assess attentional processes and working memory level exhibit good psychometric properties related to reliability and internal consistency. The reliability of the visual attention test of Menozzi et al. [12] has been measured, not in the HMD environment yet, but in the CM environment. The study of Huang et al. [33] showed that repetition of the test within a day do not show a clear effect on sensitivity d', indicating that the test has a good test-retest reliability. Overall, previous studies provided evidence that HMD can be a reliable assessment tool. In general, however, there is a scarce body of research evaluating continuous performance tools based on VR that are used for measuring attentional capacity [1]. Research has thus far produced conflicting evidence. Some studies suggested that the allocation of attentional resources in HMD may be superior to a desktop with a 2D screen [34]. Li et al. [34] compared task performance and EEG-based neural metrics captured during a perceptual discrimination task with an HMD and conventional CM. The experiments revealed enhanced selective attention abilities in HMD environment. However, there are also studies that could not demonstrate any difference between attention performance on the HMD than on the CM. For example, Aksoy et al. [35] compared the performance in the visual n-back working memory task using an HMD and a CM. Before each target or distractor stimuli, a composite cue appeared that indicated the depth and location where the stimuli would appear. There was no significant difference in reaction times and correct response rates between the two displays. Rupp et al. [36] found similar results. Participants performed a 3-choice vigilance task using a CM and an HMD. The HMD environment was similar to the real-world environment. It was shown from the perspective of a person sitting on a table where a CM was placed. The vigilance task was presented on the monitor, and the VR controllers that participants were holding were shown in front of the viewer. One controller had a green button and the other had a red button. In this way, participants could also see in the HMD environment which button they had to press for the respective response to a stimulus. There was no significant difference between the attention performance measured in both environments.

### 1.3 Aim

The objective of the present study was, on the one hand, to investigate whether the visual attention test of Menozzi et al. [12] is also applicable in the HMD, and on the other hand, to contribute to the under-researched area of using continuous performance tests based on VR. For this purpose, it was investigated whether the visual attention performance recorded with the HMD differed from the performance measured with the CM. The results of previous studies do not give a clear indication of whether the performance measured with the HMD is better, the same, or worse than the performance measured with the CM. As it was a laboratory study in a low-distraction environment without social interaction, the effects of isolation from the environment and of social inhibition are assumed to be negligible, and not contributing to significantly better performance during the test with the HMD than with the CM. Finally, we propose the following hypothesis, H1:

H1 Visual attention performance is better in the CM environment than in the HMD environment due to negative physical effects caused by the HMD, such as induced sickness or visuo-sensory impairments.

## 2 Materials and methods

### 2.1 Materials

#### 2.1.1 Visual attention test

##### 2.1.1.1 Computer monitor

**Fig. 1:**
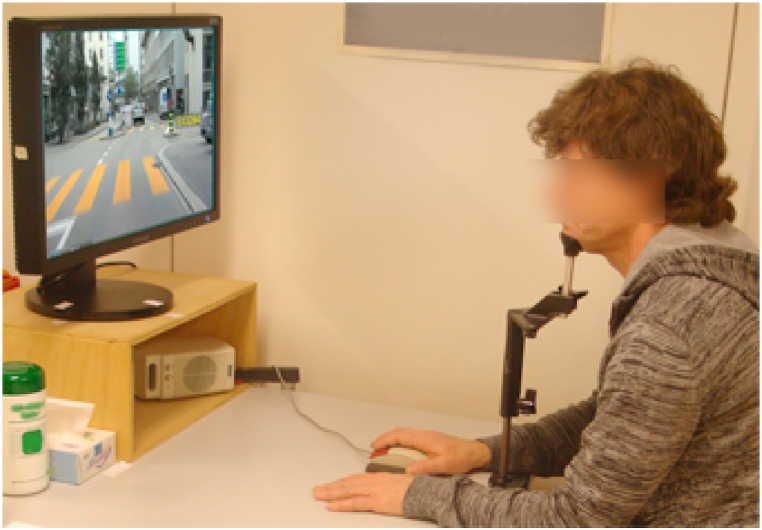
Experiment setup of visual attention test administered on a CM.

**Fig. 2:**
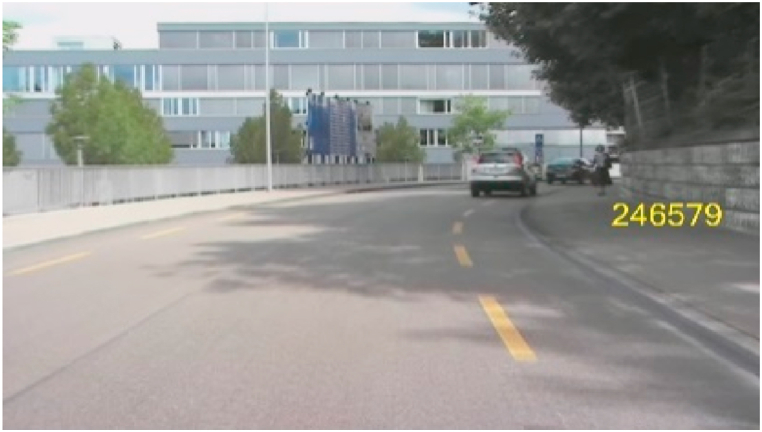
A template scene of the visual attention test showing a movie of a car drive in the background and a 6-digit number presented in the right visual field.

The former visual attention test of Menozzi et al. [12] is performed on a Samsung 245B Plus 24″ monitor, operating at a resolution of 1920 × 1200 pixels and a refresher rate of 75 Hz, which is viewed from a distance of 0.75 m. A head and chin rest is used to keep the viewing distance constant (Fig. 1). The distance considers a slightly uncorrected myopia in the average population and allows presbyops to perform the test without having to compensate for the inability of their eyes to focus on nearby objects. On the display, a background movie is presented showing a car drive from the driver’s perspective. A stimulus consisting of a six-digit number appears for 300 ms either at −15.3° (left), 0° (center), or 15.3° (right) on the horizontal meridian of the visual field (Fig. 2). The user’s task is to click the right computer mouse button marked in green if the integer ‘3’ is included in the flashed number and click the left mouse button marked in red if there is no ‘3’. Right-handed users operate the mouse with the right hand, with the red button clicked by the index finger and the green button clicked by the middle finger. Left-handed users operate the mouse with the left hand, and thus the red button with the middle finger and the green button with the index finger. The interstimulus time is set randomly and varied between 1.75 s and 4.75 s. When there is no number presented, a cross is displayed in the center which users must fixate. In total, the test consists of 72 trials and takes 4 min. The detection performance is calculated by the so-called detectability d′, which is based on the theory of signal detection [37]. D′ is the difference in the z-transform of probability for hit, occurring when the integer ‘3’ is present and the subject answered ‘yes’, and the z-transform of probability for false alarm, occurring when ‘3’ is not displayed but the participant answered ‘yes’. In addition, the response time to a stimulus is recorded. Further technical details on the attention test [are given in the paper of Menozzi et al. [12].

##### 2.1.1.2 HMD

**Fig. 3:**
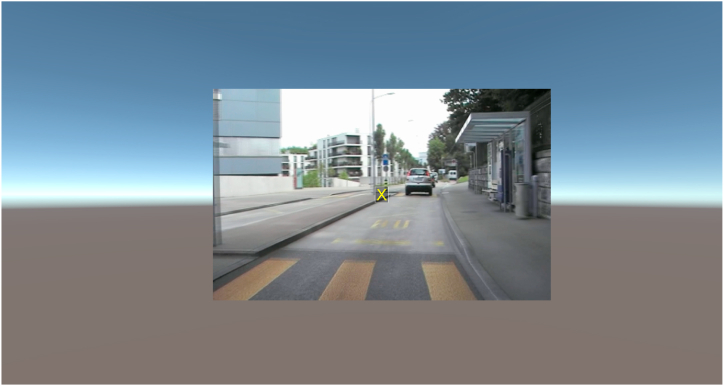
A template scene of the visual attention in the HMD environment.

The visual attention test was programmed in Unity 2019.4.3 (Unity Technologies, San Francisco, California, USA) for use in the Oculus Quest 2 HMD (Facebook Technologies) which has a screen resolution of 1832 × 1920 pixels and a refresher rate of 72 Hz (Fig. 3). The attention test is presented on an anchored 2D display at a distance of 1.3 m, as this was reported as the design focal distance for the Quest 2 [38]. The FOV that the visual attention task utilized in both HMD and computer conditions is the same so that the recorded performance is comparable. The display moves with head rotation and its size is adapted so that it is perceived as being as large as the desktop computer display at a distance of 0.75 m. The Oculus touch right hand controller is used to respond to the stimuli. The button A is marked in green, and the button B is marked in red.

#### 2.1.2 Visual acuity measurement

The Snellen E chart was used to measure visual acuity. The participant stood in front of the E chart at a distance of 6 m and held a piece of paper in front of one eye so they saw the E chart with only one eye. The participant must read aloud the letters on the chart, starting with the top line, and continue as long as they recognize the letter. If the participant fails to correctly identify at least half of the letters on the current line, the test is stopped and the last line in which the participant recognizes more than half of the letters was noted. The same procedure is repeated for the other eye. Participants wearing corrective lenses must keep them on.

#### 2.1.3 Participants

Participants must be at least 18 years old. Participants who declared to take drugs or whose visual acuity was poor (better eye: ≤0.5/poorer eye: ≤0.2) were not excluded from participation, but their results were not included in the data analysis of this study.

A total of 30 participants were recruited (11 f, 19 m), with an average age of 22.57 [SD = 2.46] years. The ages ranged from 20 to 29. All participants met the requirement for visual acuity and no participants reported being under the influence of drugs.

### 2.2 Methods

#### 2.2.1 Procedure

**Fig. 4:**
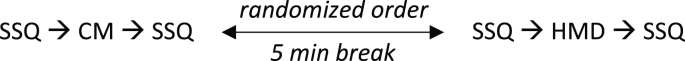
Overview of study procedure.

The experimental design was a within-subject randomized approach. Before beginning the experiment, participants were informed about the study procedure and asked to provide informed consent. At the beginning, participants completed a questionnaire about personal information, including age, gender, whether they were right- or left-handed, whether they were wearing any lenses, and if yes, whether they were near- or far-sighted. Furthermore, they were asked about their medication or drug intake in the last 24 h. Afterwards, the visual acuity was measured using the Snellen E chart. Subsequently, they performed the visual attention test on the CM and with the Oculus Quest 2 in a randomized order with a break of 5 min in between the tests (Fig. 4). During the experiment, participants sat in a quiet room at a table, on which the controller and computer mouse were placed. To familiarize themselves with the experiment, participants completed a set of 10 trials before each attention test, which was repeated as long as more than 50% of the answers were incorrect. Before the test with the Oculus Quest 2, the interpupillary distance was adapted for the participant, so that they perceived a clear image in the HMD. Participants were instructed not to move their head during the attention test with the HMD to alleviate motion sickness. Before each test, they were told to respond to stimuli as quickly and accurately as possible, even if they were unsure of the answer. As a state measurement of severity of sickness, the Simulator Sickness Questionnaire (SSQ) [39] was used. The SSQ is the most widely used measurement tool of negative side effects caused by immersions in VR, simulator sickness, and cybersickness [40,41]. The SSQ score was obtained before each test, as well as after each test, to determine whether participants were already suffering from symptoms at the beginning of the test. At the end of the experiment, they were asked to indicate which of the two tests was more difficult (forced choice: HMD/CM). In addition, they were asked to give any comments about the tests in order to gain information about the usability of the test with the HMD. The whole procedure lasted approximately 25 min. The study was not conducted at the same time for each participant.

The ethical committee of ETH Zürich (approval No 2021-N-207) approved the study protocol.

#### 2.2.2 Data analysis

The statistical analysis of the data was performed using IBM SPSS Statistics software (version 26, SPSS Inc., Chicago, IL, USA). A level of p = 0.05 was assumed for a significant impact.

##### 2.2.2.1 Repeated measures ANOVA with the within-subject factors ‘location in the visual field’ (left, center, right) and ‘test environment’ (CM, HMD)

The data analysis focused on comparing the performance in the three locations of the visual field which were obtained using the CM and the HMD. An analysis of variance (ANOVA) with repeated measures was run in which the factors ‘location in the visual field’ (three levels: left, center, right) and ‘test environment’ (two levels: CM, HMD) were considered as within-subject factors. Bonferroni adjustment was used for post-hoc pairwise comparisons between factor means. In addition, in cases where the interaction was significant, simple effects tests were performed to reveal the degree to which one factor is differentially effective at each level of the other factor. These analyses were conducted for both the detectability and response time. Partial eta square (ηp2) was used as an estimate of effect size in interpreting the ANOVA results [42].

##### 2.2.2.2 Comparison of attention performance measured in the present study and a previous study

In a second step, the data analysis focused on comparing the results of the present study to those of the age-matched group of participants (N = 35, age range: 20–29 y; 9 f, 26 m) reported in a previous study [12]. For this purpose, ANOVA was used to determine the similarities between the results of the present and previous studies, considering the location of the target in the visual field (left, center, right) as the within-subject factor and the study (present, previous) as the between-subject factor. This analysis was conducted once between the data sets measured with the CM in the present study and with the CM in the previous study, and once between the data sets recorded with the CM in the previous study and with the HMD in the present study.

##### 2.2.2.3 Simulator Sickness Questionnaire scores (SSQ scores)

**Comparison of the SSQ scores recorded after the tests with the CM and HMD**

In the last step, the SSQ scores obtained after the tests with the CM and HMD were compared using a paired-sample *t*-test to control whether participants performed the tests under the influence of significantly different sickness levels.

**Comparison of the difference in the SSQ scores recorded before and after a test between the tests with the CM and the HMD**

The difference in the total SSQ score, between the score recorded before and after a visual attention test, was compared between the attention tests performed with the CM and the HMD by means of a paired-sample *t*-test. The purpose was to test whether there was a significantly greater increase in sickness level during one of the tests than the other.

**The effect of concerning symptoms of simulator sickness and oculomotor discomfort on the attention performance**

To investigate whether simulator sickness and oculomotor discomfort negatively affected d' in the HMD, a one-way multivariate analysis of variance (MANOVA) was conducted with three dependent variables (location of signal in the visual field: left, center, right), whilst the independent variable was once “SSQ”, which consisted of two categories defined according to the suggestion that total SSQ scores can be associated with concerning symptoms when it is equal to or higher than 15 [43]: “concerning symptoms” (SSQ score ≥15) and “no concerning symptoms” (SSQ score <15), and once “oculomotor discomfort”, which included the categories “oculomotor discomfort experienced” and “oculomotor discomfort not experienced”.

## 3 Results

The ANOVA was conducted to examine the effect of the presented number’s location and the device used on d’ and the response time. Table 2 presents the ANOVA results using d’ as the criterion.

**Table 2:** ANOVA results using d' as the criterion.

Predictor	Sum of Squares	df	Mean Square	F	p-value	Effect size: ηp2
location	4.83	2	2.42	9.71	<0.01	0.25
test environment	6.15	1	6.15	19.86	<0.01	0.41
location x test environment	0.97	2	0.49	3.76	0.03	0.12

**Fig. 5:**
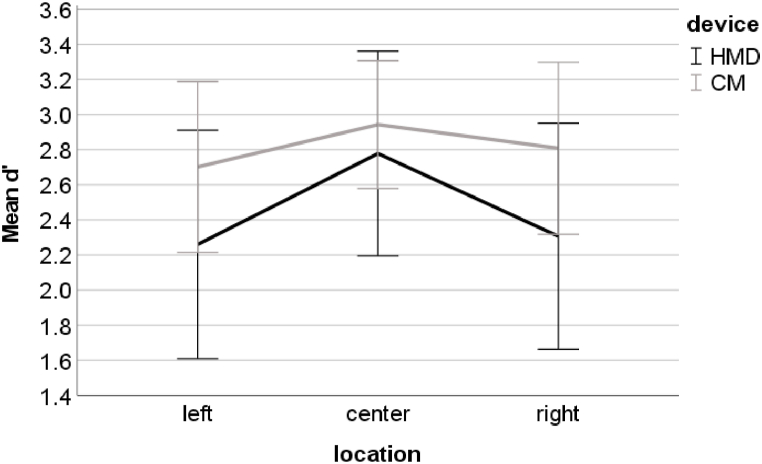
Average and one standard deviation of d′ for the task performed in the left, central, and right visual field with the HMD (black line) and with the CM (grey line). For more clarity, the scale starts at 1.4.

The performance recorded with the CM and the HMD significantly differed in the left visual field (CM: M = 2.70, SD = 0.49; HMD: M = 2.26, SD = 0.65, padjusted <0.01, 95% CI of the difference = 0.19 to 0.70) and in the right visual field (CM: M = 2.81, SD = 0.49; HMD: M = 2.31, SD = 0.65, padjusted < 0.01, 95% CI of the difference = 0.28 to 0.73) (Fig. 5). No significant difference between the two test environments was found for the d’ in the central visual field (padjusted = 0.11). Moreover, Bonferroni-adjusted comparisons indicated that, in the HMD environment, the d’ was significantly higher in the central visual field (M = 2.78, SD = 0.58) than in the left visual field (padjusted < 0.01, 95% CI of the difference = 0.23 to 0.81), and significantly higher in the central visual field than in the right visual field (padjusted < 0.01, 95% CI of the difference = 0.16 to 0.78). In contrast, for the CM environment, d′ in the left, central (M = 2.94, SD = 0.36), and right visual fields were not significantly different (left vs. central: padjusted = 0.06; left vs. right: padjusted = 1.00; right vs. central: padjusted = 0.54).

Furthermore, we found a significant difference in response time assessed in the CM (M = 781.70 ms) and HMD environments (M = 840.67 ms). The ANOVA results using response time as the criterion is presented in Table 3.

**Table 3:** ANOVA results using response time [ms] as the criterion.

Predictor	Sum of Squares	df	Mean Square	F	p-value	Effect size: ηp2
location	548153.62	2	274076.81	137.70	<0.01	0.83
test environment	156511.21	1	156511.21	9.71	<0.01	0.25
location x test environment	3497.54	2	1748.77	1.46	0.24	0.0.05

**Fig. 6:**
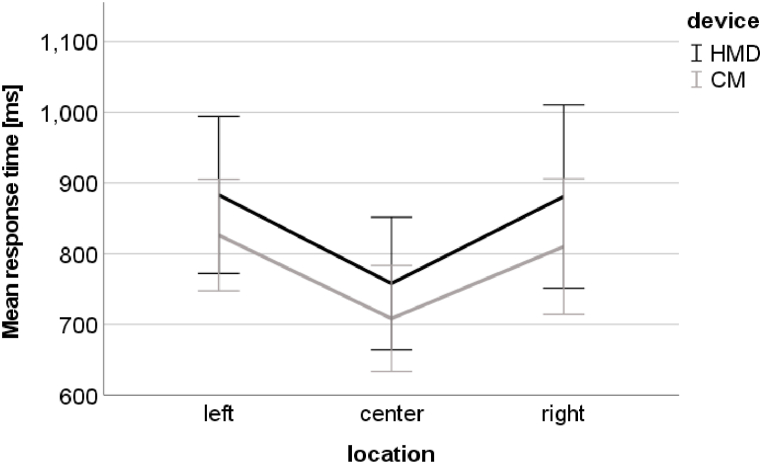
Average and one standard deviation of response time for the task performed in the left, central, and right visual field with the HMD (black line) and with the CM (grey line). For more clarity, the scale starts at 600 ms.

The response time was in the central visual field (M = 733.32 ms, SD = 87.62 ms) significantly lower than in the left (M = 854.69 ms, SD = 99.56 ms) and the right (M = 845.54 ms, SD = 118.35 ms) visual fields (padjusted < 0.01) (Fig. 6). The response time did not differ between the left and right visual fields (padjusted > 0.05).

**Fig. 7:**
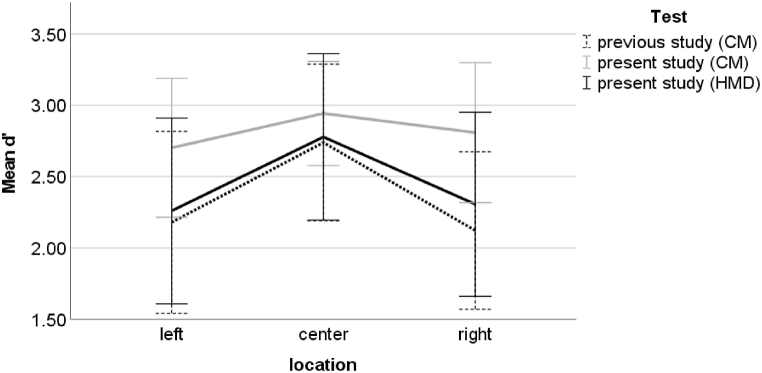
Average and one standard deviation of d’ for the task performed in the left, central, and right visual fields with the HMD (black line) and the CM (grey line) in the present study and the task performed with the CM (dashed black line) in the previous study. For more clarity, the scale starts at 1.5.

The comparison of the d’ recorded with the CM in the previous study of Menozzi et al. [12] with results of the present study recorded with the HMD did not show an effect of interaction between location of the target in the visual field and study (F (2, 126) = 0.39, p = 0.68, ηp2 = 0.01). There was also no significant main effect of study on d’ (F(1, 63) = 0.80, p = 0.38, ηp2 = 0.01). The ANOVA with the data set of the present and previous studies measured with the CM revealed a significant main effect of study on d’ (F(1,63) = 23.97, p < 0.01, ηp2 = 0.28). The performance measured in the present study was significantly higher (M = 2.82, SD = 0.46) than that which was measured in the previous study (M = 2.35, SD = 0.64). Furthermore, there was a significant interaction between the effects of location and study on d′ (F(2, 126) = 5.15, p < 0.01, ηp2 = 0.08) (Fig. 7). Bonferroni-adjusted comparisons revealed that the d′ was significantly higher in the left (M = 2.70, SD = 0.49) and right (M = 2.81, SD = 0.49) visual fields in the CM environment of the present study than in the CM environment of the previous study (left visual field: M = 2.18, SD = 0.64, padjusted < 0.01, 95% CI of the difference = 0.24 to 0.81; right visual field: M = 2.12, SD = 0.55, padjusted < 0.01, 95% CI of the difference = 0.43 to 0.95). The performance in the central visual field did not significantly differ (padjusted = 0.09).

In the present study, six participants showed some concerning SSQ scores (≥15) during the test with the HMD headset. Four of these six participants already showed a concerning SSQ score before the test with the HMD headset. Fifteen participants experienced oculomotor-related discomfort during the test with the HMD and seven of these fifteen participants complained about oculomotor-related discomfort during the test with the CM.

Only two participants reported that the test with the CM was more difficult than the test with the HMD. The SSQ score measured after the attention test was significantly higher (p = 0.01) in the test with the HMD (M = 8.85, SD = 12.79) than in the test with the CM (M = 4.36, SD = 8.80). The differences of the total SSQ score between the score before and after a visual attention test were not significant between the tests with the HMD (M_SSQbefore-after_ = −4.49) and CM (M_SSQbefore-after_ = −1.99; p = 0.27). The MANOVA revealed no significant effect of having concerned symptoms at the end of the test with the HMD or experiencing oculomotor discomfort in the HMD environment on d′ (p > 0.05). Moreover, there were no concerns regarding the usability of the HMD reported.

## 4 Discussion

This study compared the visual attention performances recorded with Menozzi et al.’s [12] test presenting targets on a conventional CM, and with an analog version of the test presented in the Oculus Quest 2 HMD. The latter device is more practical for use in occupational environments.

Results revealed that the visual attention performance was significantly lower when recorded with the HMD than with the CM, as manifested by a significantly lower d′ and significantly longer response time. This is in agreement with H1, which alleges that the performance is better when recorded with the CM than with the HMD. This finding also agrees with feedback from the majority of the participants, who stated that the test was more difficult with the HMD than with the CM.

### 4.1 Comparison of the findings with previous studies

The results are not consistent with previous studies which found either no difference between attentional performance measured with CM and HMD [35,36] or enhanced selective attention abilities in the HMD environment [34]. Different experimental setups may account for the disagreement in the studies' results. For example, in Li et al.’s (2020) study, the task included depth and location information indicating where the stimuli would appear in the visual field. The authors stated, as a potential explanation for the VR-induced enhanced attention, that HMD-VR increased the visuo-spatial sensory inputs and heightened the activation of brain areas that are important for signal processing. In the present study, these factors did not play a role as the test was presented on a 2D screen in the HMD. This may be one reason why the performance measured with the HMD was worse than that measured with the CM, in contrast to Li et al.’s study [34]. In the study by Aksoy et al. (2021), the n-back test was conducted, and the stimuli were only presented in the center of the visual field. When only considering the results in the central visual field in the present study, the results agree with Aksoy et al. (2021). In Rupp et al.’s (2019) study, participants saw the controllers with the colored marked buttons which indicated which button must be used for ‘yes, the signal is present’ and for ‘no, the signal is not present’. In the present study, participants did not see the controller and had to recall by heart which controller button was green and which was red. This may have been difficult for some of the participants, particularly because they had to answer under time pressure. This could have led to an increased response time, as participants first had to think about which controller button to press. That possibly resulted in more missed signals—meaning participants were too slow to respond to one stimulus until the next appeared—and also in more incorrect responses, as cognitive capacity may have been reduced and thus there was less capacity to focus on target detection. These are possible explanations for why, in the present study, unlike in Rupp et al.’s (2019) study, the performance measured with the CM was better than the performance measured with the HMD. A future study should investigate how the visibility of the controller affects visual attention performance. For this purpose, visual attention performance recorded once with the HMD version of this study and once with another HMD version in which the controller with the colored marked buttons is presented in VR should be compared.

### 4.2 Possible causes of lower attention performance on the head-mounted display than on the computer monitor

As predicted, the lower performance with the HMD could be caused by oculomotor discomfort, which was observed in half of the participants, while only eight participants showed oculomotor discomfort when performing the test with the CM. Acute visual symptoms, such as blurred vision, eyestrain, and difficulty focusing and concentrating are assumed to increase the likelihood of incorrectly detecting stimuli [30,31]. However, the results showed that participants who experienced oculomotor discomfort did not show worse performance than those who did not experience any oculomotor discomfort. Another negative side effect of HMDs which could influence attention performance is sickness induced by exposure to VR. The SSQ scores assessed after the tests were generally higher when the test was performed with the HMD than with the CM. It must be emphasized that the SSQ score determined before the test with the HMD was already significantly higher than the values determined before the test with the CM. This could have led to the performance differences in the tests with the CM and the HMD. However, when comparing performances during the test with the HMD between participants with concerning and no concerning sickness symptoms, there were no significant performance differences. Conclusively, the results give no evidence that the performance in the HMD environment is worse than in the CM environment due to increased oculomotor discomfort or sickness. However, an important finding for using the HMD in the practice is that there was no significant difference between the devices in the difference calculated from the SSQ scores measured before and after each test. This indicates that HMD does not cause more sickness than the conventional CM. Conclusively, the results support H1 in regards to the statement that performance is better when measured with the CM than with the HMD, but not regarding the cause of the difference. A potential reason for the lower attentional performance during the test with the HMD could be extraneous indicators. For instance, the test on the HMD is a new experience for some people, and this may lead to a depletion of cognitive resources rather than contributing to performing [44]. However, to minimize this effect, participants performed a test trial to familiarize themselves with the test environment. Another reason for increased cognitive demands in the HMD environment was previously discussed; namely, the possibility of reduced attention performance because participants did not see the controller, resulting in an increased demand on the cognitive load in the test with the HMD. This means that less cognitive resources are available for the attention task. Hence, possible increased cognitive load during the test with the HMD may explain a lower performance when using the HMD as compared to the CM.

Regarding the longer response time in the test with the HMD, the possible influence of system latency of the HMD device should also be considered. The term latency is considered as the sum of all latencies across components that go from the generation of a signal until it reaches the human [45]. Gruen et al. (2020) measured the system latency of the Oculus Quest 1 with a hardware instrumentation-based measurement method that yielded a system latency of 81 ± 2.5 ms. If this latency were subtracted from the response time measured during the HMD test in the present study, it would result in an average response time of 759.70 ms, which is a shorter response time than that measured during the test using the CM (M = 781.70 ms). If the Oculus Quest 1’s system latency is similar to Oculus Quest 2, it could be assumed that the response time in the test environments does not differ significantly. However, further investigations will have to be conducted to assess the latency measured during the visual attention test in the Oculus Quest 2. For this purpose, a study similar to that of Gruen et al. could be conducted in the future.

### 4.3 Possible causes of different levels of attention performance across the visual field

A difference in attentional performance was found in the central and peripheral visual fields. One explanation is the increased cognitive load which can shrink the visual field and cause tunnel vision, resulting in better performance in the central visual field [46]. Regarding response time to the stimuli, significant differences were observed between the performance in the center and in the periphery in both tests, whereas, regarding the signal detection, only the performance during the test with the HMD showed a significant difference between the central and peripheral visual fields. This is in contrast to a previous study of Menozzi et al. [12] in which a significant difference between the central and peripheral visual fields were shown in the test with the CM. However, the performance on the CM in the present study showed a tendency to be better in the central visual field than in the periphery. This better performance in the center visual field is consistent with previous studies [12,47]. A reason other than tunnel vision caused by increased cognitive demand for the difference in central and peripheral performance can be the increase in noise, caused by the complex visual background, with the size of the visual field in the attention test resulting in a higher resource requirement to process irrelevant information in the periphery than in the center. Consequently, there are less cognitive resources available for processing peripheral targets than central targets [13]. Performance may also vary across the visual field due to physiological factors, such as the correlation between cortical magnification and visual acuity, which is the highest in the fovea [48,49], as well as due to cognitive factors. According to Wickens et al.’s [50] SEEV (salience, effort, expectancy, and value) model of attention states that physical or mental effort is required in order to change current behavior to attend areas of interest and assess available information. In the context of the visual attention test, effort is defined as the extent to which participants must move their eyes to access information but also to filter out the relevant information from the irrelevant. The physical effort is higher in the periphery because eye movements to peripheral targets are more extended than when the target appears in the center. Conclusively, the effort is higher when allocating attention to the periphery, meaning that more cognitive load is required in the periphery compared with the center.

A reason for the greater difference between peripheral and central d’ measured in the HMD environment than in the CM environment could be the center-bias effect that occurs with sickness induced by VR [21]. As six participants experienced concerning symptoms during the test with the HMD, a center-bias could occur and lead to an extended difference between the performance in the periphery and in the center. However, the results did not reveal any significant effect of simulator sickness on d’ measured during the test with the HMD. Therefore, it can be assumed that another reason causes the greater difference. It is worth noting that the d’ recorded during the test with the HMD behaves similar to the d′ measured during the visual attention test on the CM, in which the task’s cognitive load increased by shortening the presentation time of the stimuli to 200 ms (see Fig. 1 in Ref. [7]). This indicates that the cognitive load is increased in the HMD, which is in accordance with the participants' perceptions of difficulty. The increased cognitive load reduces available cognitive resources that may lead to a stronger tunnel vision effect during the test with the HMD compared to the test with the CM. The less available cognitive resource during the test with the HMD may still be sufficient for detecting central targets as smaller effort is required than for detecting peripheral targets. Conclusively, it seems that the visual attention task is more difficult when using the HMD than when using the CM environment, especially in the peripheral visual field. This may lead to a more sensitive detection of the visual skill of high performers. Regarding response time to the stimuli, the differences between the central and peripheral response times were similar in both tests (Fig. 1B). An explanation for the similar behavior of the response time across the visual field in both tests could be that participants tended to respond as quickly as possible to stimuli even if they were unsure about the correct answer, and therefore, they did not hesitate long in responding to stimuli in the periphery.

### 4.4 Comparison of the results of the present study with previous studies on the visual attention test of Menozzi et al

Finally, it must be emphasized that the CM performance measured in the present study was significantly different from the performance measured with CM in the previous study [12]. The reason the performance on the CM measured in the previous and present studies significantly differed might be due to the different environmental conditions. The risk of environmental bias could be reduced by a more standardized test.

The HMD environments meet the requirement of a highly standardized assessment condition to ensure reliable measurement. Previous studies [1,20,32] showed sufficient reliabilities of assessment of attentional processes with VR tools. Related to Menozzi et al.’s visual attention test, Huang et al. [33] who provide indication of good test-retest reliability when using CM. Based on the findings of those studies and the fact that the settings are better controlled in HMD environment than in CM environment it is assumed that the visual attention test is also reliable when using the HMD. To support this assumption, the reliability of the visual attention test with the HMD will be investigated in a future study.

The suitability of Menozzi et al.’s test [12] for measuring attention performance with the HMD was supported by the fact that no participant reported any concerning problems regarding the usability when performing the test with the HMD. In addition, no problems with the functionality of the HMD were noted.

### 4.5 Limitation

A limitation of this study is that only young people between 20- and 29-years-old were tested. In a next step, the same study will be conducted but with older participants. The difference between the performance measured using HMD and CM is expected to be larger than in the present study, since older people are generally less acquainted with HMDs, and thus have reservations about it and might need more time for familiarization than younger people [51]. In addition, older people’s cognitive capacities are reduced, and age-related vision problems occur, influencing visual performance.

Moreover, the study was conducted in a laboratory that did not correspond to a usual work environment. Consequently, the influence of auditory and visual distractions from the work environment and the influence of social inhibitions from other people, which was theoretically expected to cause a better performance when recorded with the HMD than with the CM, were not considered in this study. Due to the great advantage of the HMD being isolated from the environment, it is expected that the difference between the performance measured with the HMD and the performance measured with the CM will decrease, as it is assumed that the performance on the CM will decrease due to environmental distraction and social inhibition. The influence of social inhibition on test performance must be kept in mind for the decision about using a CM or an HMD for performance testing until further studies provide more evidence on the effect of inhibition. The assumption will be investigated in a further study by conducting the tests in occupational settings.

## 5 Conclusion

An objective assessment tool to detect workers with low attention levels would be a preventive countermeasure against occupational accidents caused by inattention. Menozzi et al.’s [12] visual attention test has been demonstrated to have several advantages over other continuous performance tests in measuring attention performance in occupational settings. In the scope of this work, the visual attention test, administered originally on a CM, was implemented into the HMD Oculus Quest 2 for more practical use in working environments. To test whether the new HMD version also effectively measures attention capacity, it was evaluated whether the visual attention performance measured with the Oculus Quest 2 HMD differed from the performance measured with the CM. The hypothesis that the attention performance is better in the CM environment than in the HMD environment could be confirmed, whereas the reason for this difference in performance could not be clearly elucidated. This leads to the assumption that a new normative data set must be collected for the HMD version. However, since the comparison between the normative data set recorded on a CM in the study by Menozzi et al. [12] and the attention performance measured with the HMD in the present study did not reveal a significant difference, this needs to be further investigated in a future study.

Furthermore, the results indicate that the test in the HMD environment generally does not cause concerning oculomotor discomfort or sickness. There were no problems regarding the functionality and usability revealed when using the HMD. This supports the suitability of the HMD version. In addition, there are several great advantages of the use of HMDs over CM environments. Utilizing HMDs' attention performance assessments improves standardization across various testing environments, as the visual stimuli are exclusively dependent on programmed stimuli. The portable HMDs facilitate assessment as the application does not require much preparation time, space, or controlling of the environmental influences. This allows for use in environments with difficult conditions.

Conclusively, it is recommended to continue research with the Oculus Quest 2. The study demonstrated that the visual attention test with the HMD has the potential to be implicated for measuring cognitive processes in occupational environments in order to detect changes in visual attention performance and can therefore contribute to a safer work environment. However, this study provides only initial insights into the suitability of the HMD version of the visual attention test for recording attention. The study serves as a good basis for future studies investigating the use of the HMD to measure attentional performance. In the next steps, the reliability of the HMD version should be examined in particular, and the suitability of HMDs in recording attention performance should be tested in elderly people.

## Funding

Tanja Baertsch reports financial support was provided by The Zurich Cantonal Police, Switzerland.

## Author contribution statement

Tanja Baertsch: Conceived and designed the experiments; Analyzed and interpreted the data; Wrote the paper.

Ying-Yin Huang: Performed the experiments; Contributed reagents, materials, analysis tools or data.

Marino Menozzi: Contributed reagents, materials, analysis tools or data.

## Data availability statement

Data will be made available on request.

## Declaration of competing interest

The authors declare that they have no known competing financial interests or personal relationships that could have appeared to influence the work reported in this paper
