# Supplementary material for: Head-mounted display versus computer monitor for visual attention screening: A comparative study
Source: Heliyon. 2023 Jun 1;9(6):e16610. doi: 10.1016/j.heliyon.2023.e16610 (PMC10360570; doi:10.1016/j.heliyon.2023.e16610)
Supplement: Multimedia component 1 [file mmc1.pdf]

Date: ________________   **Time: ___________**

ID computer: ________

ID VR: ________

Demographic Questionnaire

1. Age: ______ years
2. Gender: ☐ Male ☐ Female ☐ Diverse
3. Are you wearing any lenses now? ☐ Nothing ☐ Glasses / Contact lenses
4. You see without correction…

   near objects: ☐ clearly ☐ blurry

   distant objects: ☐ clearly ☐ blurry
5. Do you have a visual limitation or an eye disease?

   ☐ yes: ____

   ☐ no
6. Have you taken any medication or drugs in the last 24 hours? **Exception:** do not mention the contraceptive pill

   ☐ yes ☐ no

   If *yes,* which?

   Name: ________________________

   How often? ☐ regularly ☐ exceptionally only today

   Name: ________________________

   How often? ☐ regularly ☐ exceptionally only today

   Name: ________________________

   How often? ☐ regularly ☐ exceptionally only today
7. You are…
   a. ☐ right-handed
   b. ☐ left-handed

Visual acuity (*filled out by the conductor of the study*)

Left eye: _____ (line number on E chart)   Right eye: _____ (line number on E chart)

Simulator Sickness Questionnaire t0

Do you experience the following symptoms now?

	None	Slight	Moderate	Severe
General discomfort				
Fatigue				
Headache				
Eye strain				
Difficulty focusing				
Increased salivation				
Sweating				
Nausea				
Difficulty concentrating				
Fullness of head				
Blurred vision				
Dizzy (eyes open)				
Dizzy (eyes closed)				
Vertigo				
Stomach awareness				
Burping				

Simulator Sickness Questionnaire t1

Do you experience the following symptoms now?

	None	Slight	Moderate	Severe
General discomfort				
Fatigue				
Headache				
Eye strain				
Difficulty focusing				
Increased salivation				
Sweating				
Nausea				
Difficulty concentrating				
Fullness of head				
Blurred vision				
Dizzy (eyes open)				
Dizzy (eyes closed)				
Vertigo				
Stomach awareness				
Burping				

Simulator Sickness Questionnaire t2

Do you experience the following symptoms now?

	None	Slight	Moderate	Severe
General discomfort				
Fatigue				
Headache				
Eye strain				
Difficulty focusing				
Increased salivation				
Sweating				
Nausea				
Difficulty concentrating				
Fullness of head				
Blurred vision				
Dizzy (eyes open)				
Dizzy (eyes closed)				
Vertigo				
Stomach awareness				
Burping				

Simulator Sickness Questionnaire t3

Do you experience the following symptoms now?

	None	Slight	Moderate	Severe
General discomfort				
Fatigue				
Headache				
Eye strain				
Difficulty focusing				
Increased salivation				
Sweating				
Nausea				
Difficulty concentrating				
Fullness of head				
Blurred vision				
Dizzy (eyes open)				
Dizzy (eyes closed)				
Vertigo				
Stomach awareness				
Burping				

Simulator Sickness Questionnaire t4

Do you experience the following symptoms now?

	None	Slight	Moderate	Severe
General discomfort				
Fatigue				
Headache				
Eye strain				
Difficulty focusing				
Increased salivation				
Sweating				
Nausea				
Difficulty concentrating				
Fullness of head				
Blurred vision				
Dizzy (eyes open)				
Dizzy (eyes closed)				
Vertigo				
Stomach awareness				
Burping				

Which test was more difficult: ☐ Computer / ☐ VR

Any comments:

____

____

____
